# Supplementary material for: Synthesis of thia-Michael-Type Adducts between Naphthoquinones and N-Acetyl-L-Cysteine and Their Biological Activity
Source: Molecules. 2022 Sep 1;27(17):5645. doi: 10.3390/molecules27175645 (PMC9457610; doi:10.3390/molecules27175645)
Supplement: Supplementary file 1 [file molecules-27-05645-s001.zip › molecules-1876580-supplementary.pdf]

Synthesis and biological activity of thia-Michael-type adducts between naphthoquinones and *N*-acetyl-*L*-cysteine

Gabriele Micheletti[1]*, Carla Boga[1], Chiara Zalambani[2], Giovanna Farruggia[2], Erika Esposito[3], Jessica Fiori[3], Nicola Rizzardi[2], Paola Taddei[4], Michele Di Foggia[4], and Natalia Calonghi[2]*

[1] Department of Industrial Chemistry 'Toso Montanari', Alma Mater Studiorum - Università di Bologna Viale Del Risorgimento 4, 40136 Bologna Italy; gabriele.micheletti@unibo.it (GM), carla.boga@unibo.it (CB).

[2] Department of Pharmacy and Biotechnology, University of Bologna, Bologna (Italy); natalia.calonghi@unibo.it (NC), zalachiara@hotmail.it (CZ), nicola.rizzardi2@unibo.it (NR).

[3] Department of Chemistry 'G. Ciamician', Alma Mater Studiorum - Università di Bologna, via Selmi 2, 40126 Bologna (Italy); erika.esposito8@unibo.it (EE), jessica.fiori@unibo.it (JF).

[4.] Department of Biomedical and Neuromotor Sciences, Alma Mater Studiorum - Università di Bologna, via Irnerio 48, 40126 Bologna (Italy); paola.taddei@unibo.it (PT), michele.difoggia.2@unibo.it (MF).

^{1}HNMR spectrum of compound **8** Page 1

^{13}CNMR spectrum of compound **8** Page 2

ESI-MS spectrum of compound **8** Page 3

^{1}HNMR spectrum of compound **9** Page 4

^{13}CNMR spectrum of compound **9** Page 5

ESI-MS spectrum of compound **9** Page 6

^{1}HNMR spectrum of compound **10** Page 7

^{13}CNMR spectrum of compound **10** Page 8

ESI-MS spectrum of compound **10** Page 9

^{1}HNMR spectrum of compound **11** Page 10

^{13}CNMR spectrum of compound **11** Page 11

ESI-MS spectrum of compound **11** Page 12

^{1}HNMR spectrum of compound **12** Page 13

^{13}CNMR spectrum of compound **12** Page 14

ESI-MS spectrum of compound **12** Page 15

Direct infusion mass spectra of the reaction mixtures with GSH of compound **8** Page 16

Direct infusion mass spectra of the reaction mixtures with GSH of compound **9** Page 17

Direct infusion mass spectra of the reaction mixtures with GSH of compound **11** Page 18

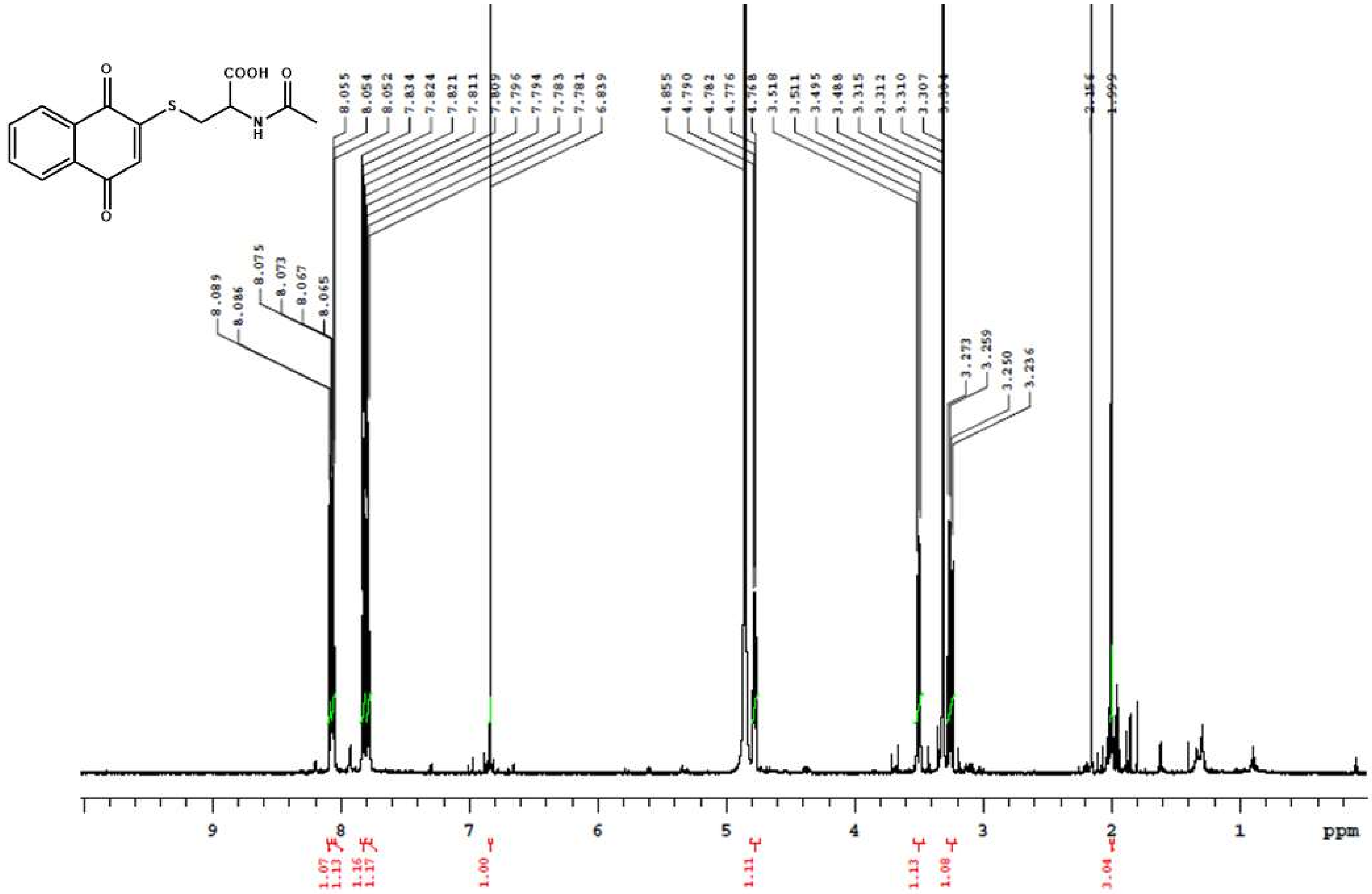

Figure S1. ^{1}H NMR spectrum in CD_3OD of compound **8**.

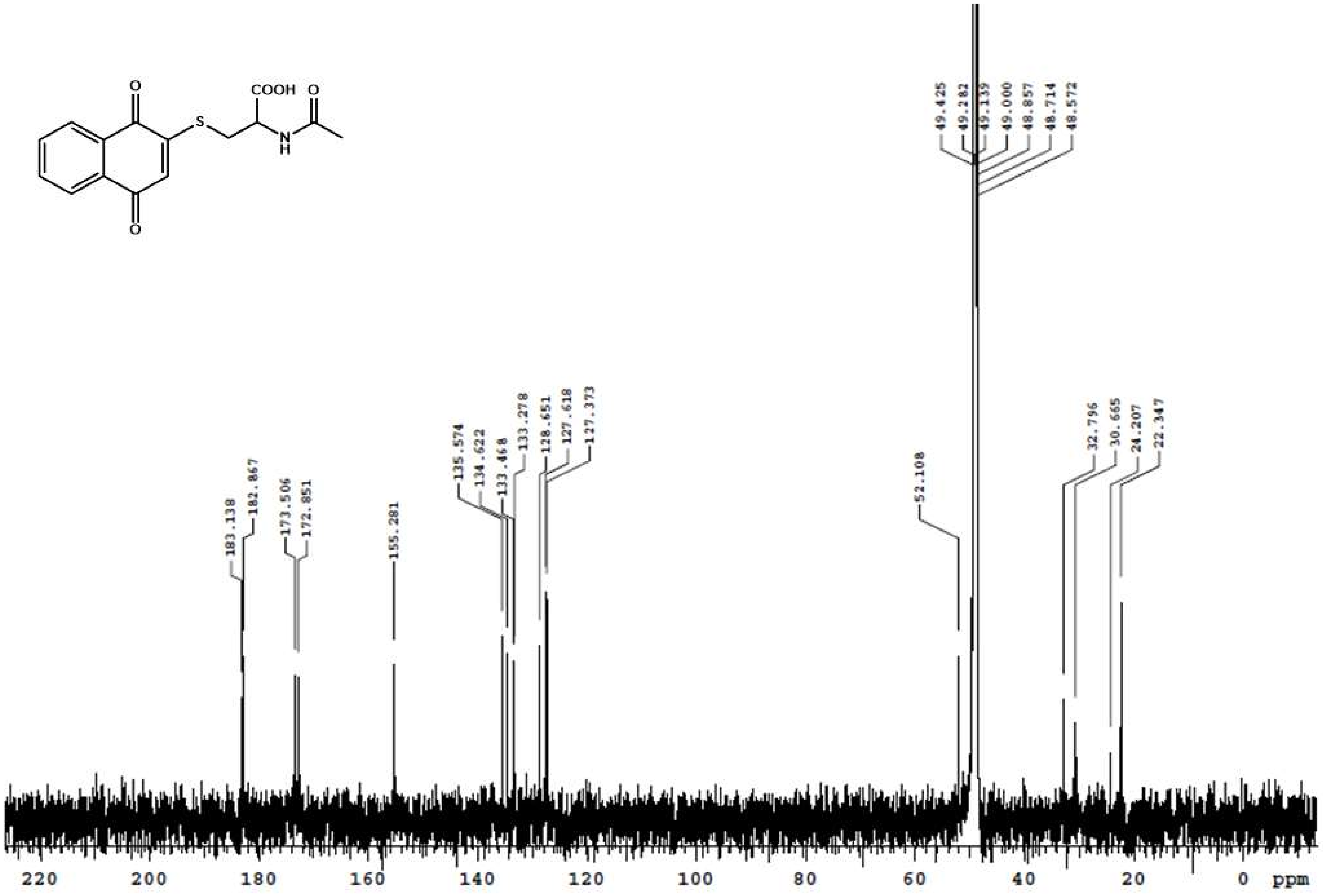

Figure S2. ^{13}C NMR spectrum in CD_3OD of compound **8**.

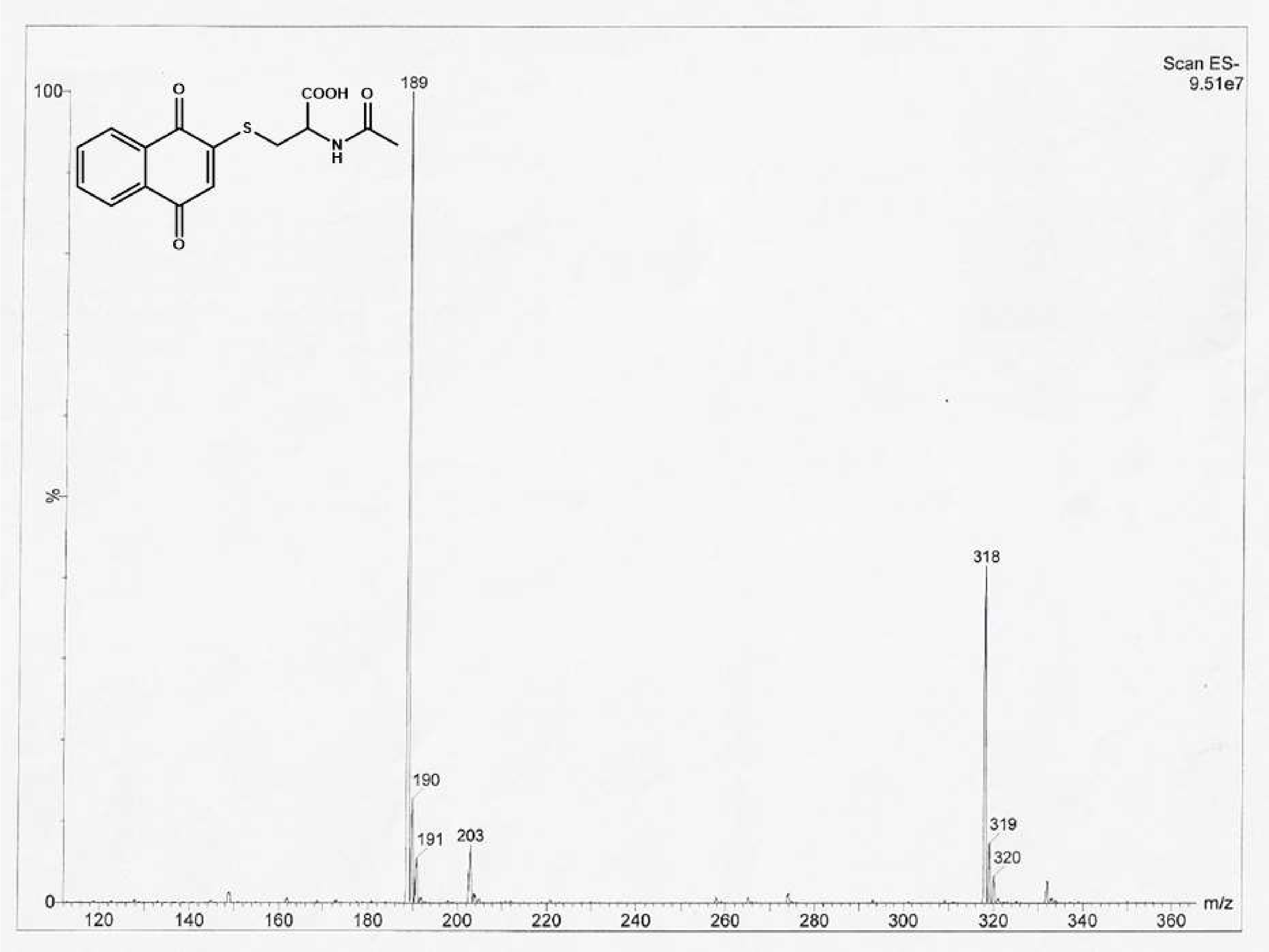

Figure S3. ESI-MS⁻ spectrum of compound **8**.

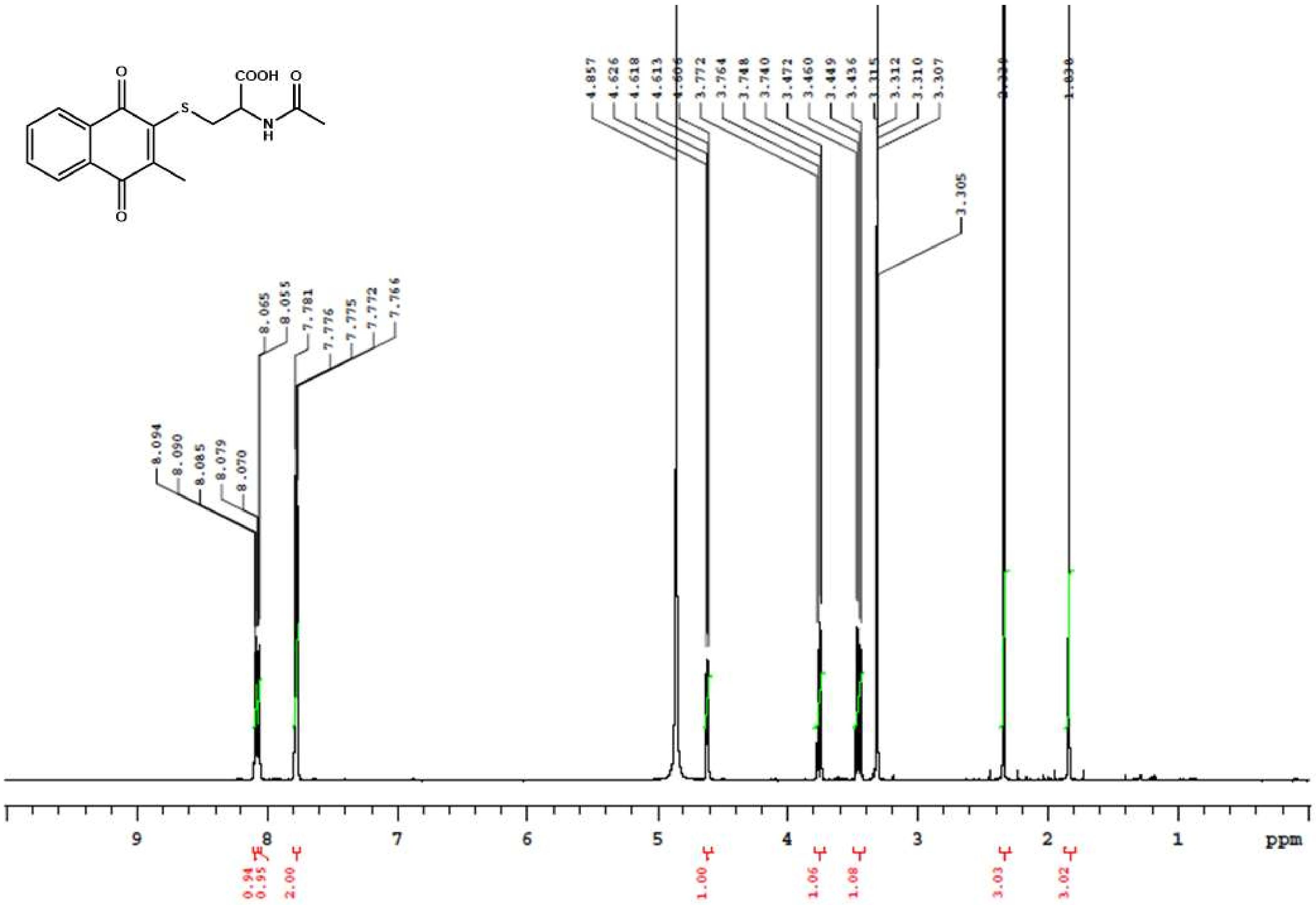

Figure S4. ^{1}H NMR spectrum in CD_3OD of compound **9**.

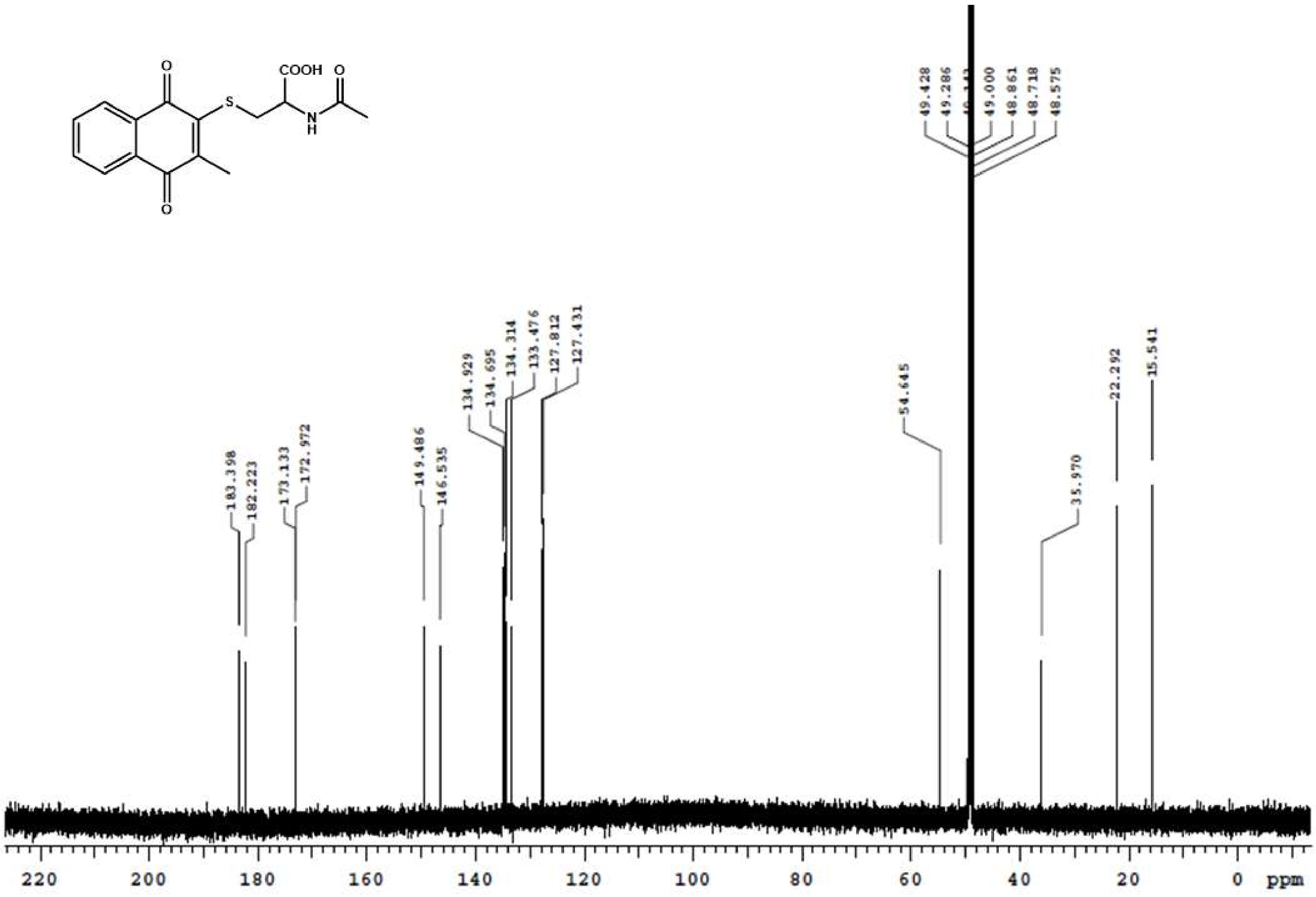

Figure S5. ^{13}C NMR spectrum in CD_3OD of compound **9**.

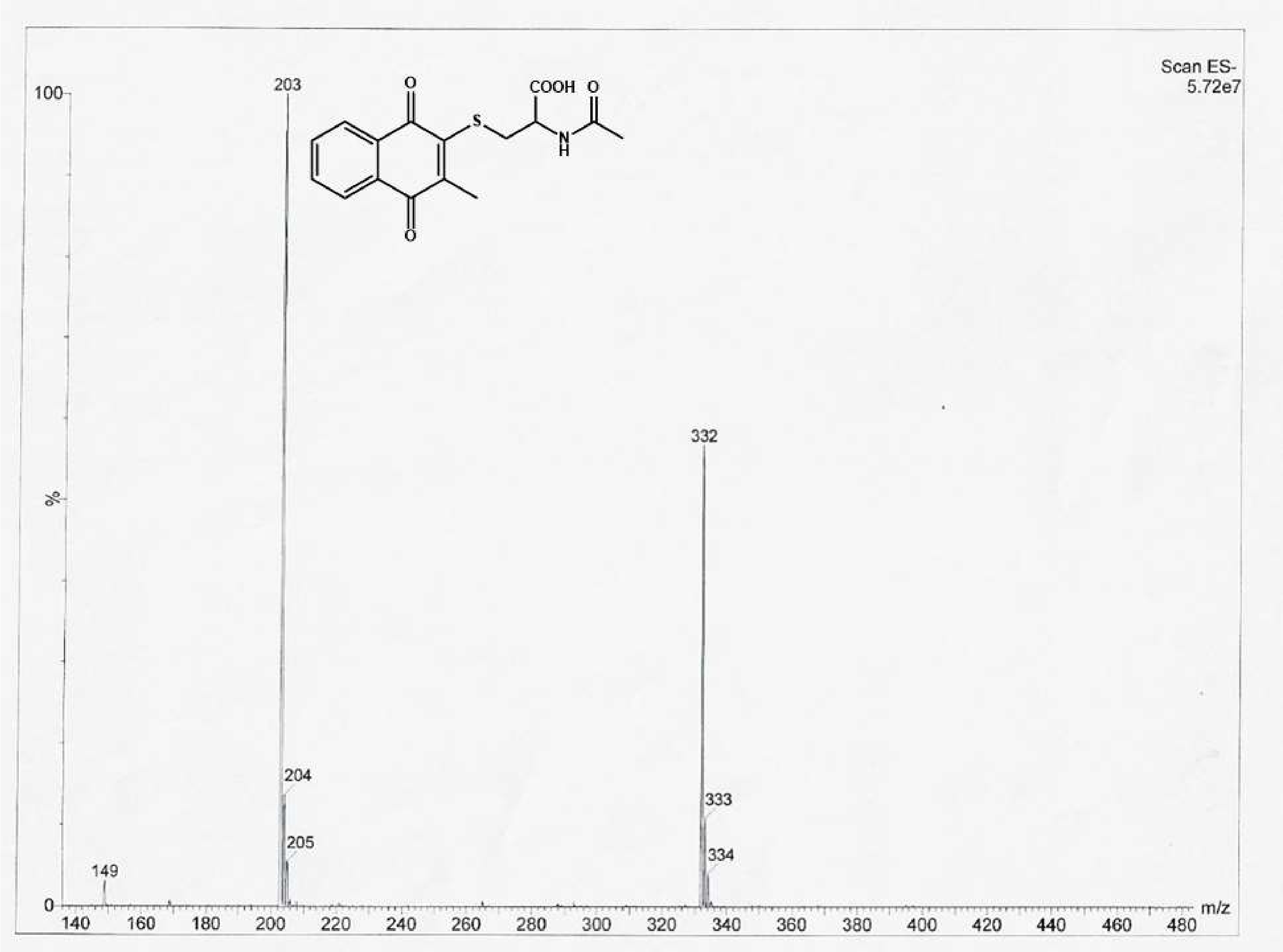

Figure S6. ESI-MS$^-$ spectrum of compound **9**.

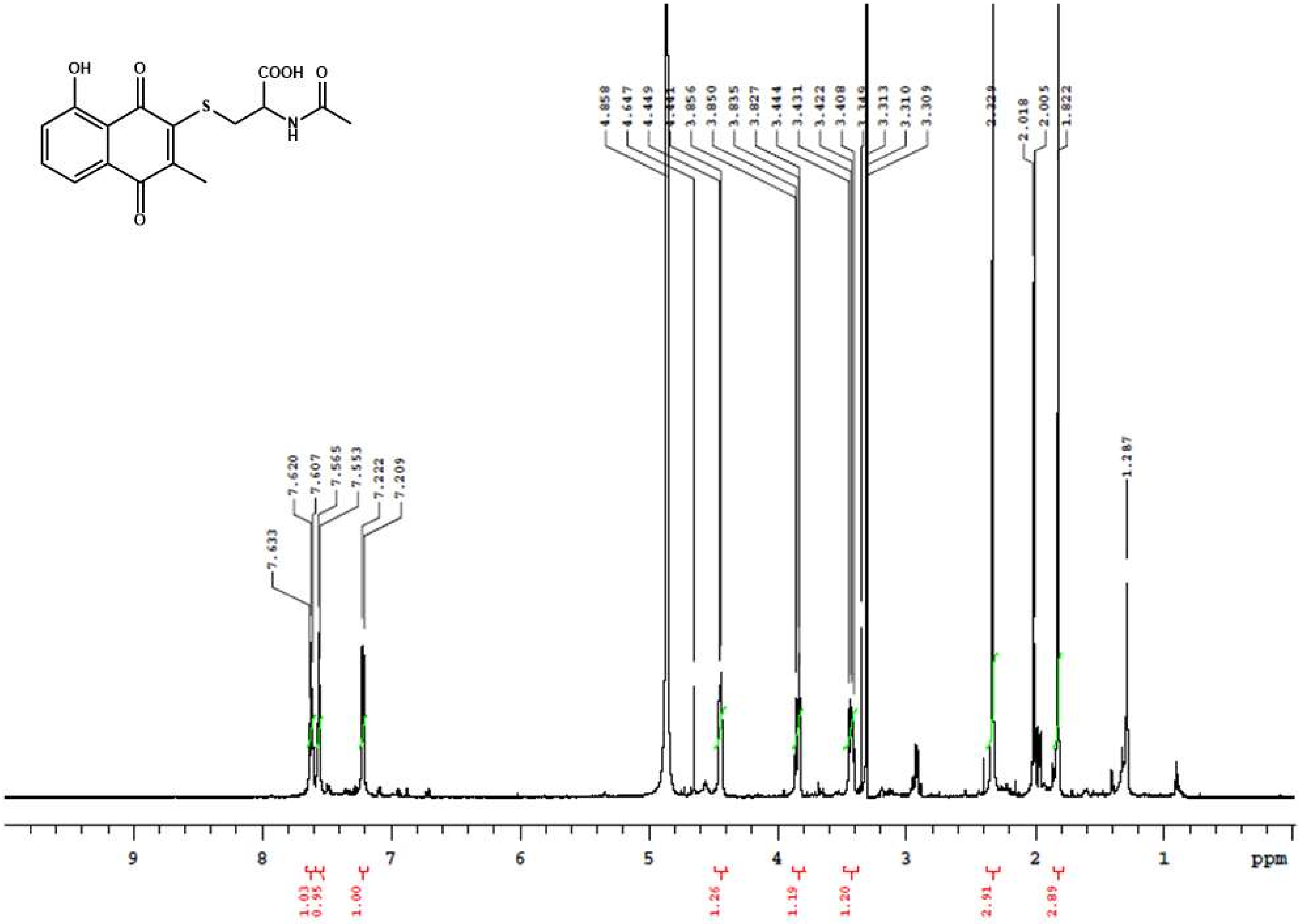

Figure S7. ^{1}H NMR spectrum in CD_3OD of compound **10**.

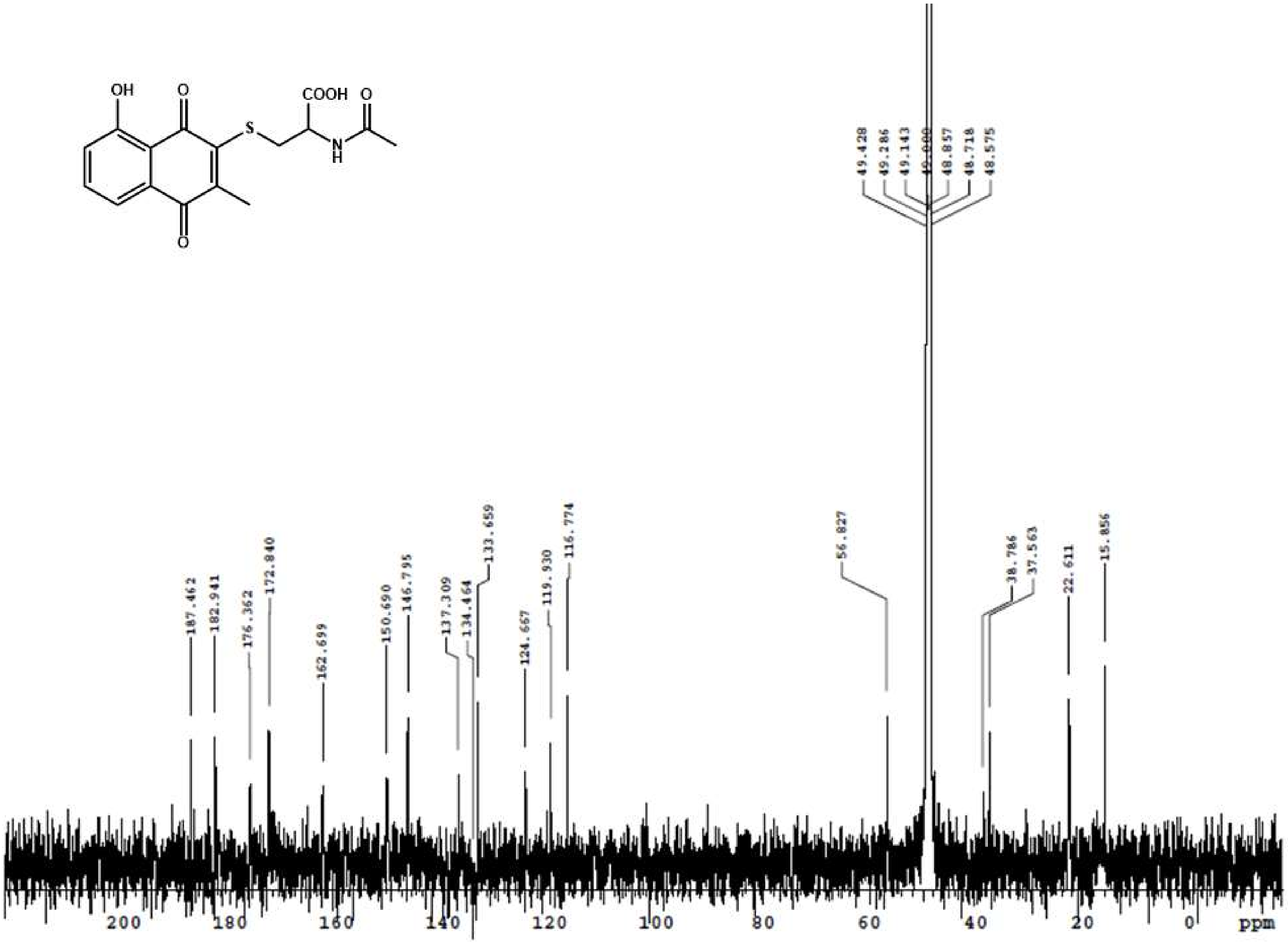

Figure S8. ^{13}C NMR spectrum in CD_3OD of compound **10**.

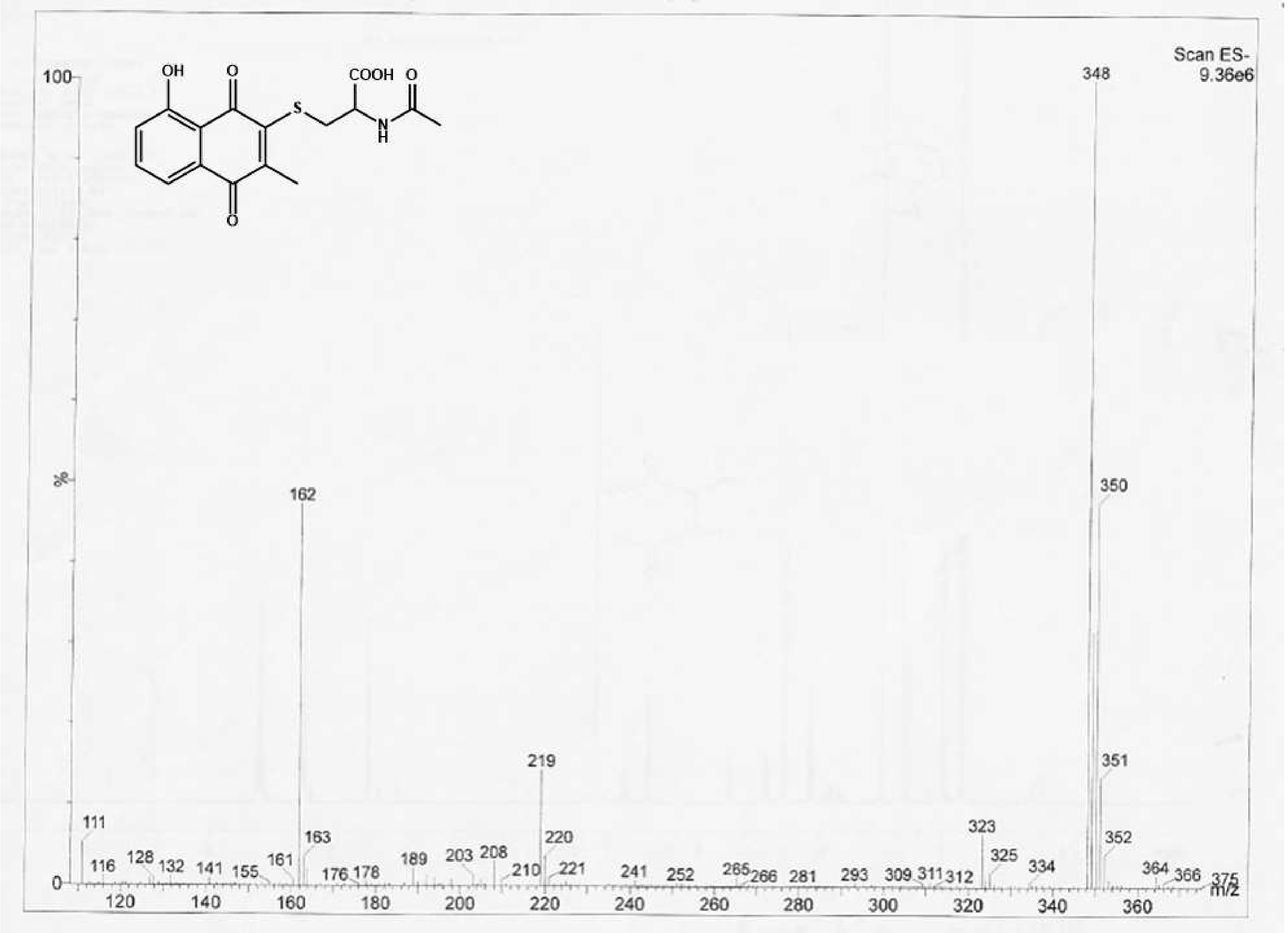

Figure S9. ESI-MS⁻ spectrum of compound **10**.

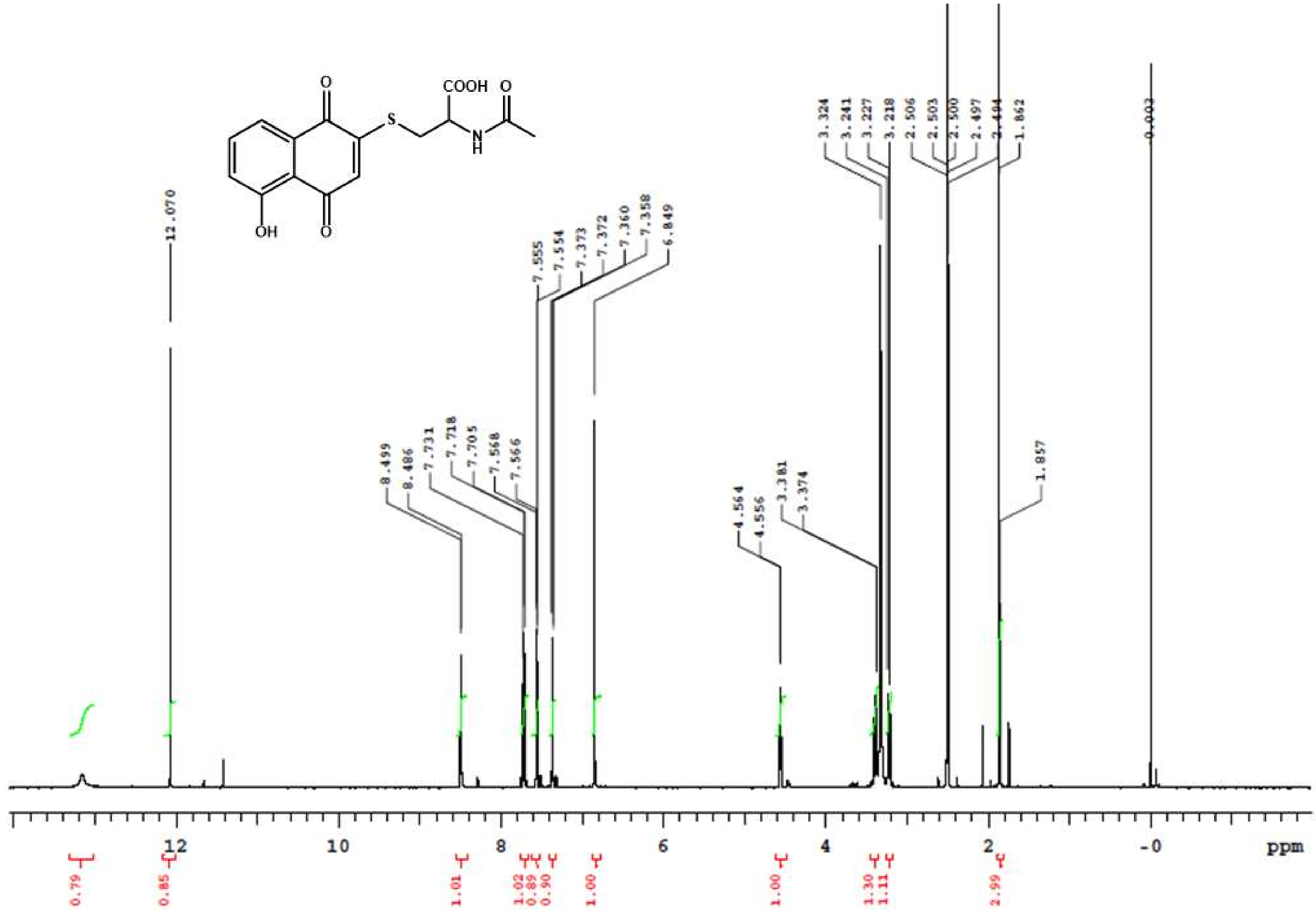

Figure S10. ^{1}H NMR spectrum in DMSO-d$_6$ of compound **11**.

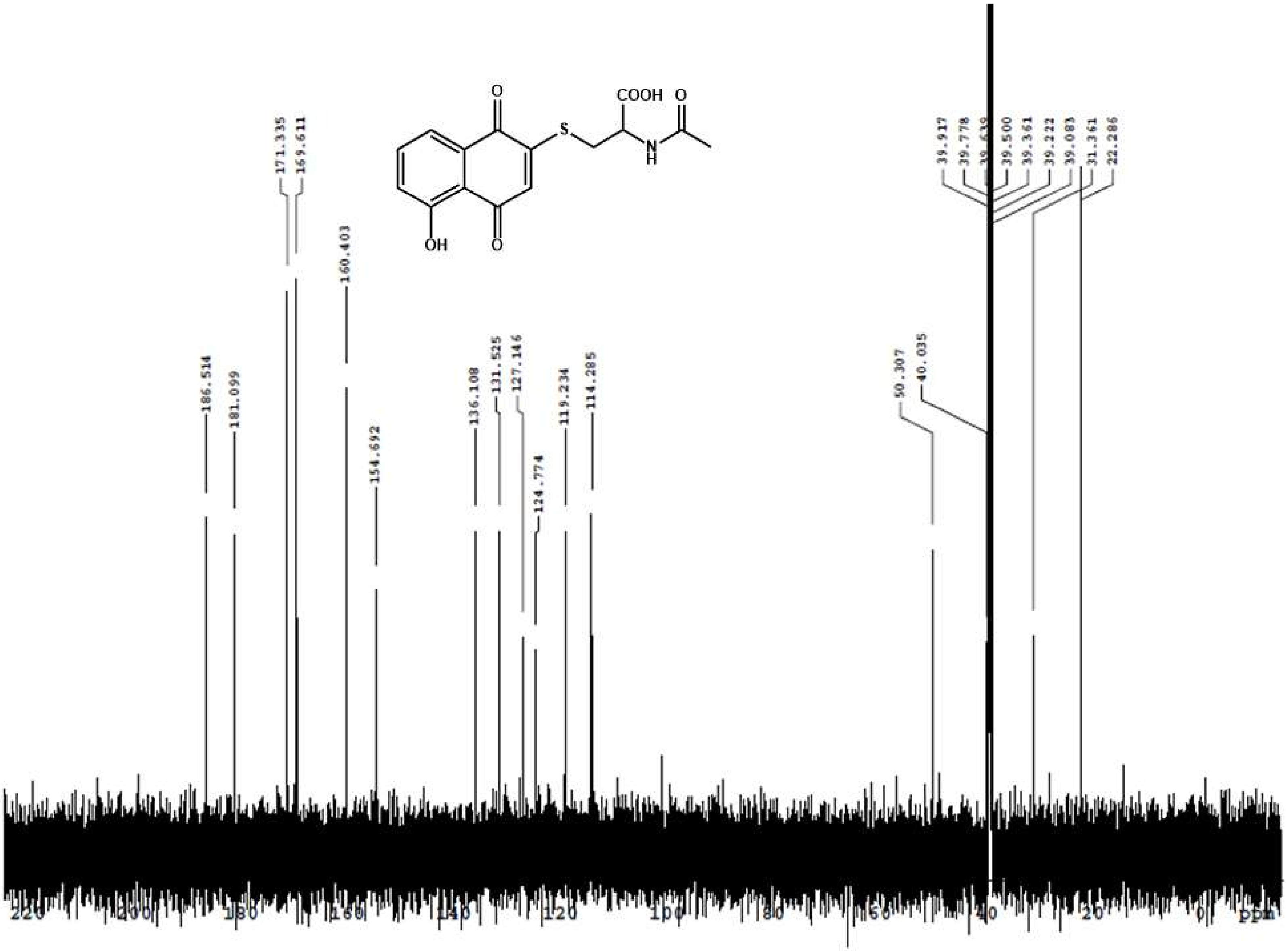

Figure S11 ^{13}C NMR spectrum in DMSO-d_6 of compound **11**.

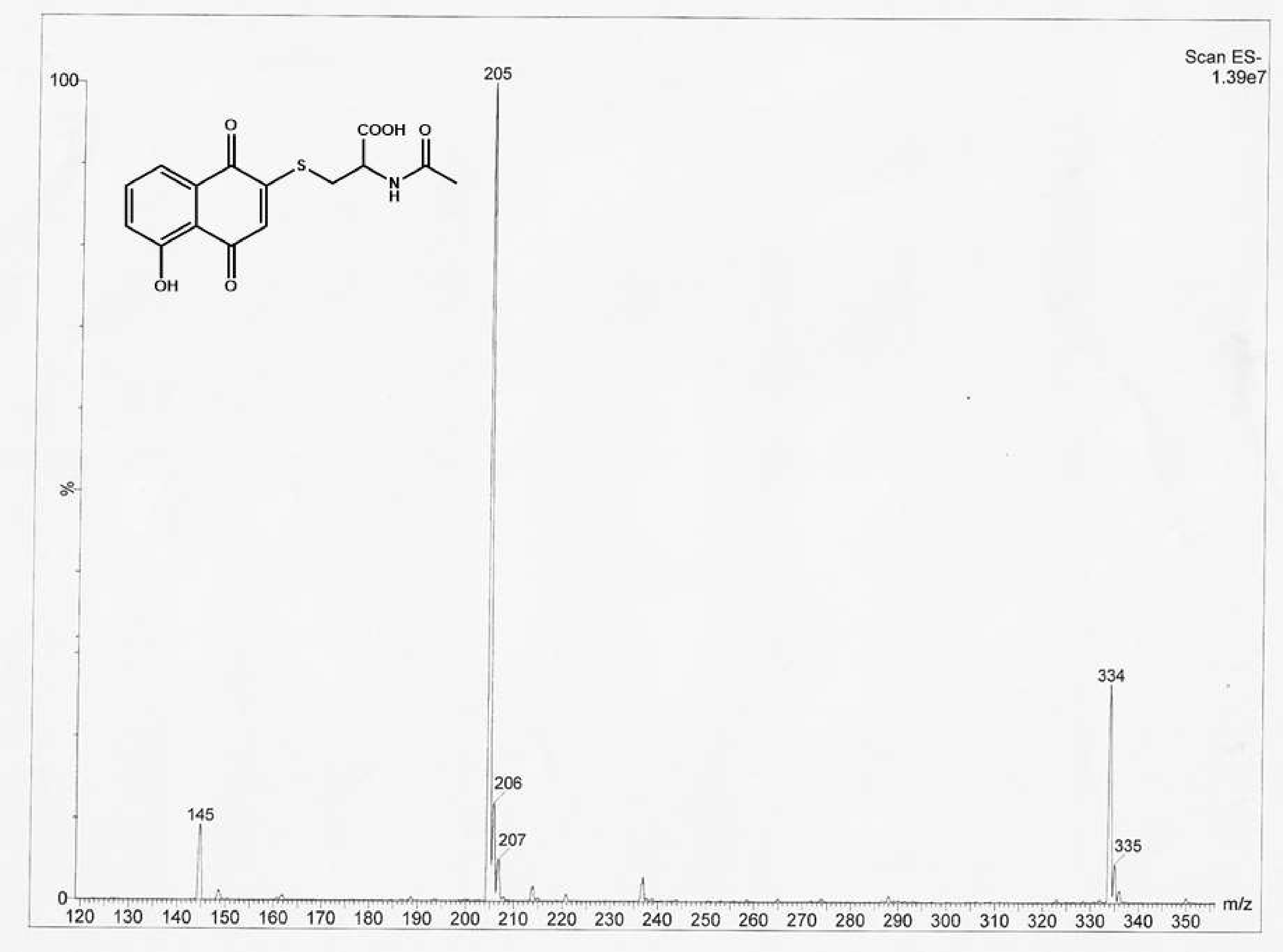

Figure S12. ESI-MS⁻ spectrum of compound **11**.

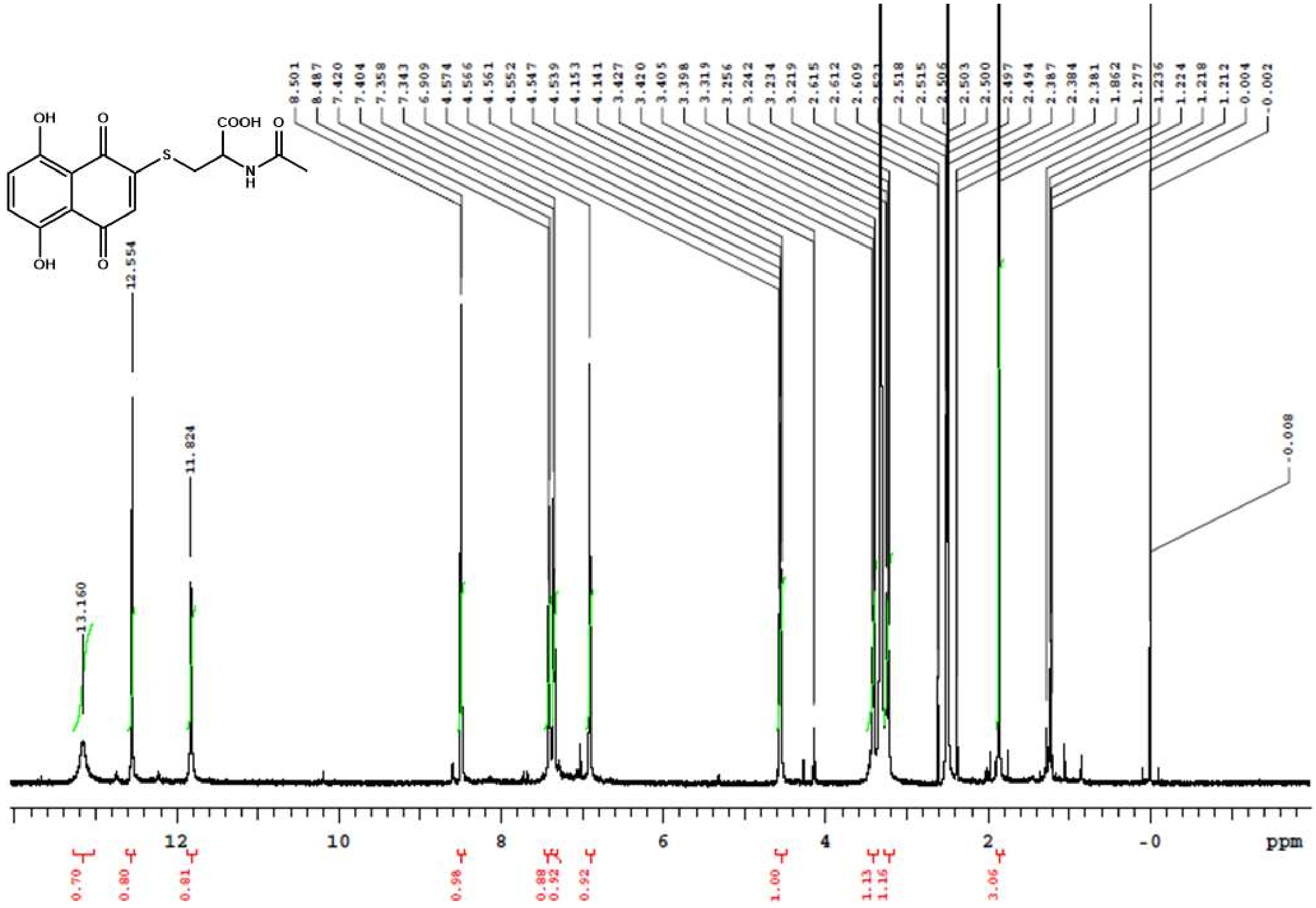

Figure S13. ^{1}H NMR spectrum in DMSO-d_6 of compound **12**.

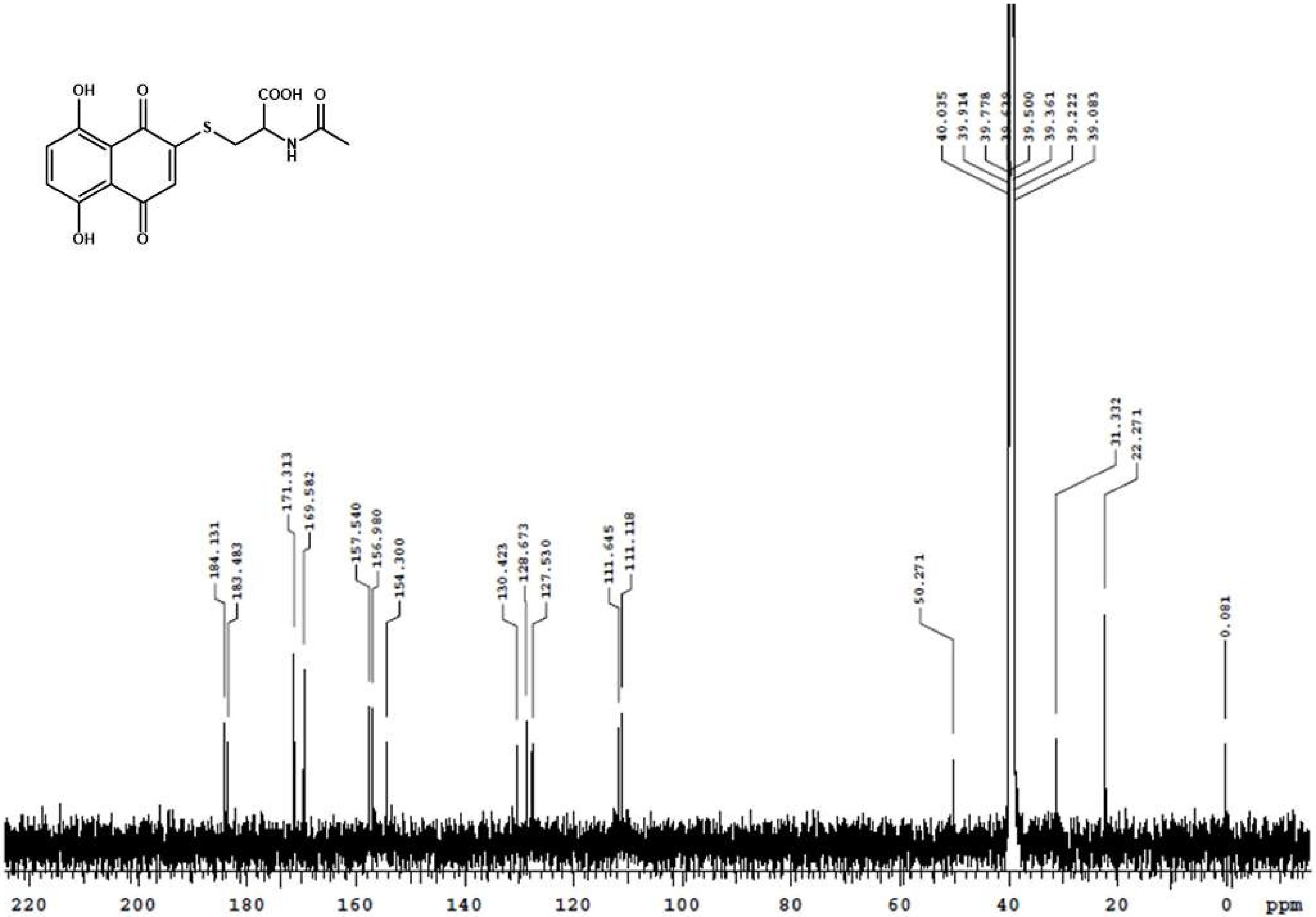

Figure S14. ^{13}C NMR spectrum in DMSO-d_6 of compound **12**.

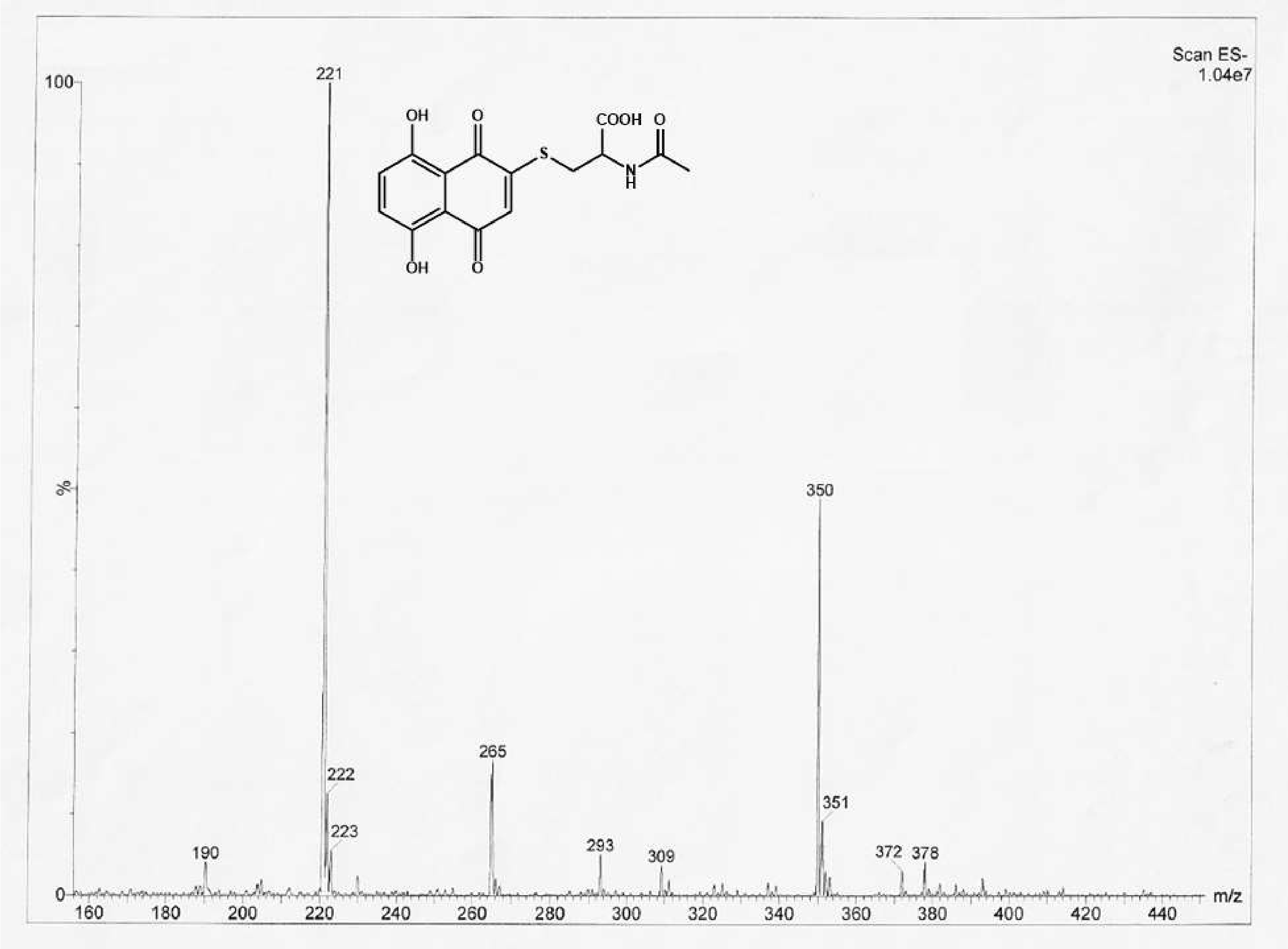

Figure S15. ESI-MS⁻ spectrum of compound **12**.

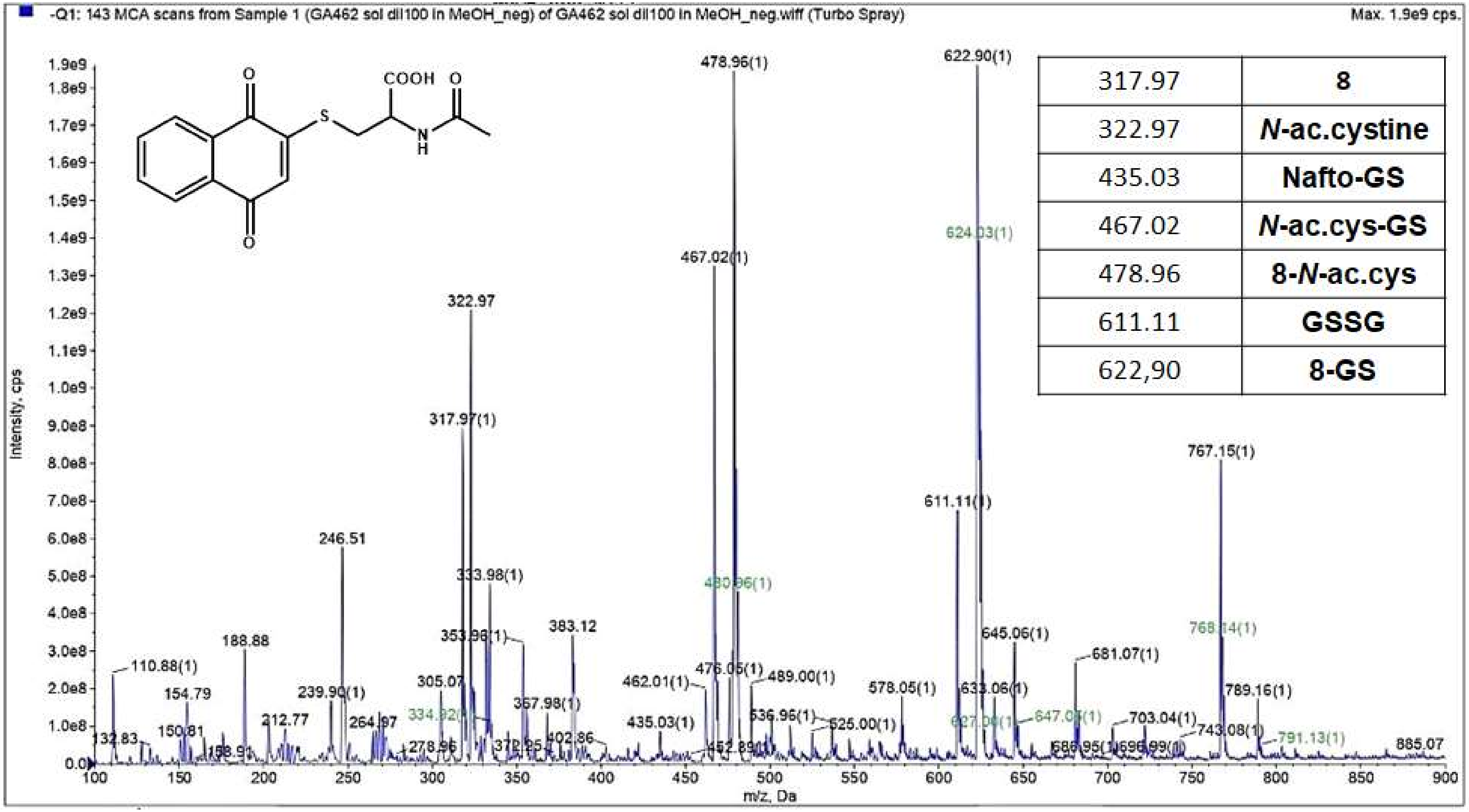

Figure S16. Direct infusion mass spectra of the reaction mixtures with GSH of compound **8**. All the detected ions are in the deprotonated form [M-H]⁻. Abbreviations: 8-GS is the adduct of **8** with glutathione, 8-*N*-ac.cys is the adduct of **8** with *N*-acetyl-*L*-cysteine, Nafto-GS is the adduct of naphthoquinone (**1**) and glutathione, GSSG: glutathione dimer; *N*-ac.cystine is the *N*-acetyl-*L*-cysteine dimer; *N*-ac.cys-GS is the *N*-acetyl-*L*-cysteine – glutathione heterodimer.

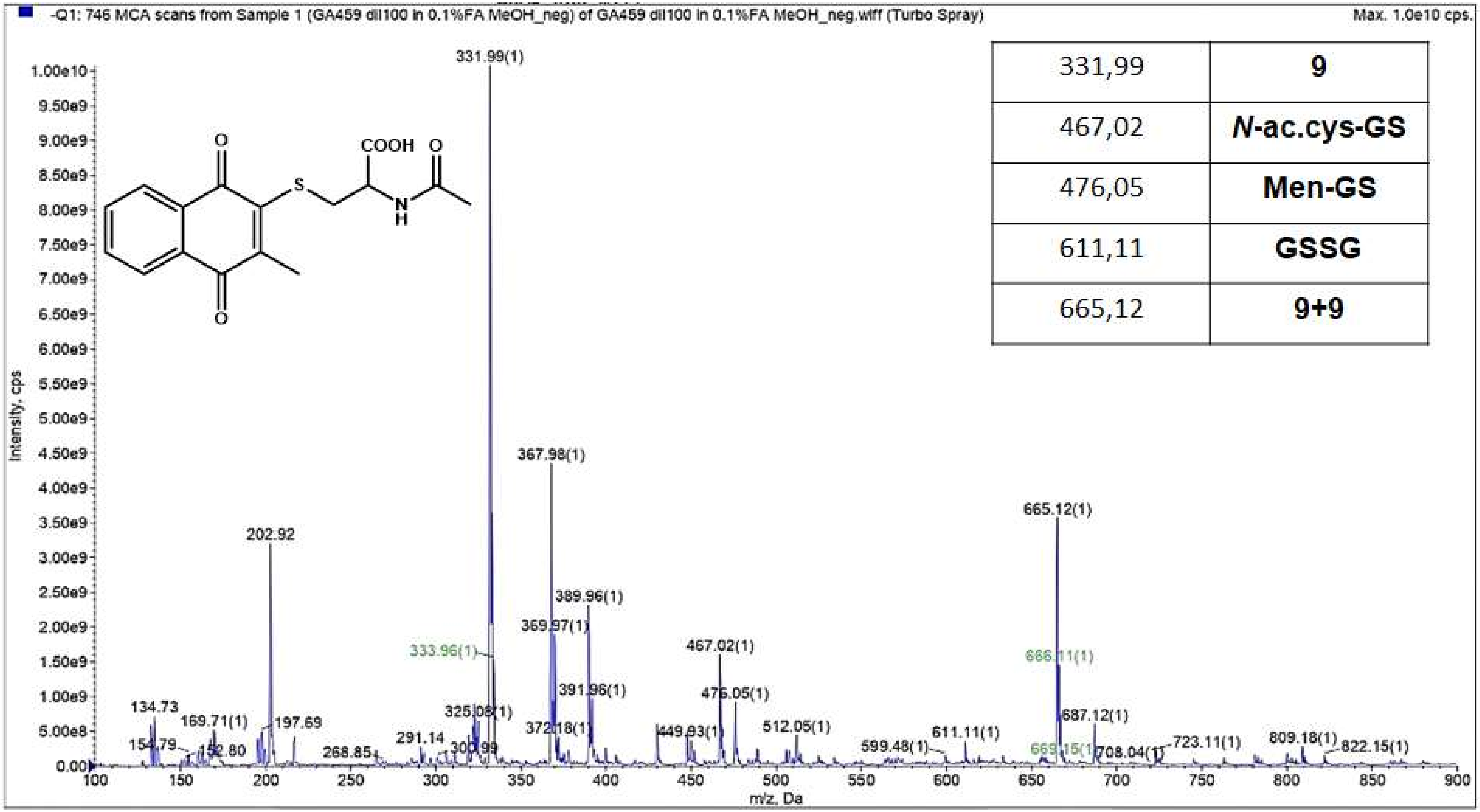

Figure S17. Direct infusion mass spectra of the reaction mixtures with GSH of compound **9**. All the detected ions are in the deprotonated form [M-H]$^-$. Abbreviations: Men-GS is the adduct of Menadione (**2**) and glutathione, GSSG: glutathione dimer, *N*-ac.cys-GS is the *N*-acetyl-*L*-cysteine – glutathione heterodimer. 9+9 is the cluster of compound **9**.

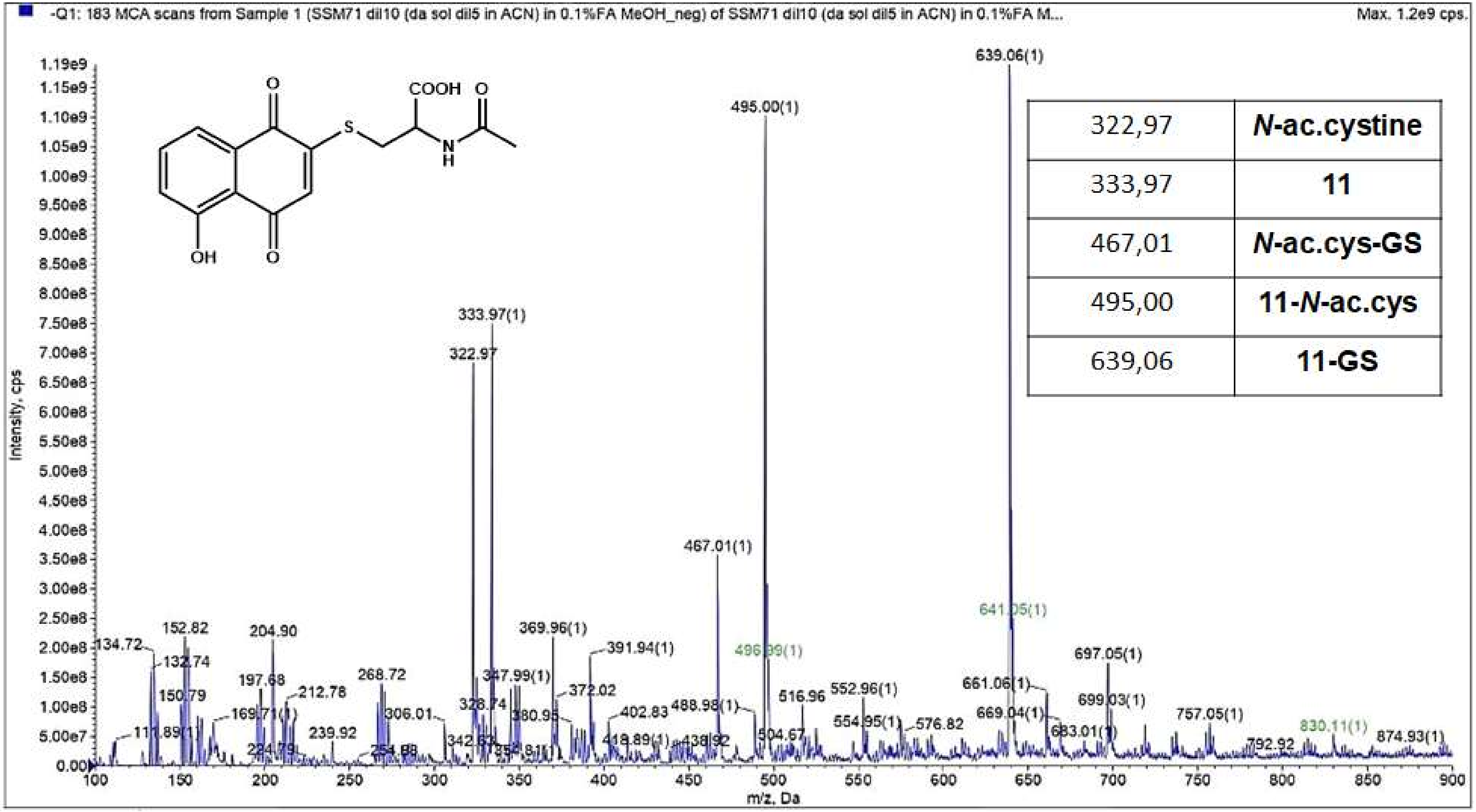

Figure S18. Direct infusion mass spectra of the reaction mixtures with GSH of compound **11**. All the detected ions are in the deprotonated form [M-H]⁻. Abbreviations: 11-GS is the adduct of **11** with glutathione, 11-*N*-ac.cys is the adduct of **11** with *N*-acetyl-*L*-cysteine, *N*-ac.cystine is the *N*-acetyl-*L*-cysteine dimer; *N*-ac.cys-GS is the *N*-acetyl-*L*-cysteine – glutathione heterodimer.
